# Air temperature suitability for *Plasmodium falciparum* malaria transmission in Africa 2000-2012: a high-resolution spatiotemporal prediction

**DOI:** 10.1186/1475-2875-13-171

**Published:** 2014-05-03

**Authors:** Daniel J Weiss, Samir Bhatt, Bonnie Mappin, Thomas P Van Boeckel, David L Smith, Simon I Hay, Peter W Gething

**Affiliations:** 1 Department of Zoology, Spatial Ecology and Epidemiology Group, Tinbergen Building, University of Oxford, South Parks Road, Oxford, UK; 2 Malaria Research Institute & Department of Epidemiology, Johns Hopkins Bloomberg School of Public Health, Baltimore, MD, USA; 3 Department of Ecology and Evolutionary Biology, Princeton University, Princeton, NJ, USA; 4 Fogarty International Center, National Institutes of Health, Bethesda, MD, USA

**Keywords:** Temperature suitability, *Plasmodium falciparum*, Africa

## Abstract

**Background:**

Temperature suitability for malaria transmission is a useful predictor variable for spatial models of malaria infection prevalence. Existing continental or global models, however, are synoptic in nature and so do not characterize inter-annual variability in seasonal patterns of temperature suitability, reducing their utility for predicting malaria risk.

**Methods:**

A malaria Temperature Suitability Index (TSI) was created by first modeling minimum and maximum air temperature with an eight-day temporal resolution from gap-filled MODerate Resolution Imaging Spectroradiometer (MODIS) daytime and night-time Land Surface Temperature (LST) datasets. An improved version of an existing biological model for malaria temperature suitability was then applied to the resulting temperature information for a 13-year data series. The mechanism underlying this biological model is simulation of emergent mosquito cohorts on a two-hour time-step and tracking of each cohort throughout its life to quantify the impact air temperature has on both mosquito survival and sporozoite development.

**Results:**

The results of this research consist of 154 monthly raster surfaces that characterize spatiotemporal patterns in TSI across Africa from April 2000 through December 2012 at a 1 km spatial resolution. Generalized TSI patterns were as expected, with consistently high values in equatorial rain forests, seasonally variable values in tropical savannas (wet and dry) and montane areas, and low values in arid, subtropical regions. Comparisons with synoptic approaches demonstrated the additional information available within the dynamic TSI dataset that is lost in equivalent synoptic products derived from long-term monthly averages.

**Conclusions:**

The dynamic TSI dataset presented here provides a new product with far richer spatial and temporal information than any other presently available for Africa. As spatiotemporal malaria modeling endeavors evolve, dynamic predictor variables such as the malaria temperature suitability data developed here will be essential for the rational assessment of changing patterns of malaria risk.

## Background

The importance of temperature on numerous components of the malaria transmission cycle has been recognized for over 100 years [1-3], and been quantified in a series of progressively more sophisticated models [4]. Meanwhile, calibration of key temperature-dependent parameters, such as those capturing effects on mosquito survivorship and extrinsic incubation period (sporogony) has been refined by laboratory experiment [5,6] and field observation [7-9]. One motivation for investigating temperature effects has been to incorporate them in spatially distributed models in order to map metrics of malaria risk across continental or global scales [10-17]. Recent studies [18,19] have combined climatic datasets with mechanistic models that link temperature to transmission intensity to produce spatially and temporally gridded estimates of temperature suitability for malaria transmission. Such outputs can then be incorporated in risk models either as empirical covariates or as spatially distributed biological variables [15].

The importance of using realistic seasonal and diurnal temperature cycles within these models rather than simpler annual or monthly mean values has been demonstrated [20,21], and these elaborations have been incorporated to varying degrees in existing temperature suitability maps [18,19]. Common to these studies, however, is reliance upon synoptic climatic data representing the average seasonal pattern, typically based on annual temperature time-series averaged across numerous years of measurements. These synoptic products have been favored because, in averaging across time, the data gaps, erratic measurements and other errors associated with temporally disaggregated (asynoptic) temperature measurements can be mitigated [22]. This aggregation comes at a cost, however: in reality, temperature patterns in a given region can vary substantially from year to year – reflecting random fluctuation, cyclical climatic events, or long-term secular trends – and this inter-annual variation is not captured by synoptic data. Synoptic representations of malaria temperature suitability are adequate when used in spatial malaria risk models that are, themselves, essentially synoptic in nature (i.e., their focus is on assessing long-term spatial patterns of risk). Increasingly, however, the rationale for modeling malaria risk is to examine or predict changes in the patterns of risk through time [23]. This makes synoptic handling of temperature effects less appropriate [24] and the incorporation of inter-annual trends and variation becomes more important: whether the intention is to investigate the association between temperature and transmission, or simply to control for it when investigating other factors of interest.

This study builds on the temperature suitability model proposed by Gething *et al*. [18] and presents the first asynoptic model of malaria temperature suitability, applied to *Plasmodium falciparum* across the African continent. This modeling endeavor spans the period from 2000 to 2012, which aligns with the recent period of major investment in malaria control [25-27]. The analysis is based on a newly produced spatiotemporal data ‘cube’ derived from satellite temperature measurements and subject to a novel nested space-time interpolation algorithm to identify, remove, and replace erroneous and missing values without the need for temporal aggregation (personal communication with Weiss DJ, Atkinson PM, Bhatt SJ, Mappin BJ, Hay SI, Gething PW). Additionally, a new model is presented here to convert observed land-surface temperatures into predictions of ambient air temperature, which is more relevant to malaria transmission [3]. The remaining manuscript describes the construction and validation of this model, presents the resulting spatiotemporal predictions, and discusses their utility in studying the contributions of temperature to recent changes in African malaria endemicity.

## Methods

This section describes the data, model construction and validation, and processing steps undertaken to generate the spatiotemporal predictions of temperature suitability. First, the input satellite-derived land surface temperature imagery is described, along with the steps undertaken to clean and validate those data to provide a robust space-time data product. Second, the statistical approach for conversion of land-surface temperature to air temperature is presented. Third, the additional temporal interpolation of these data to approximate realistic diurnal and seasonal temperature time-series is explained. Fourth, the propagation of these time-series through the Gething *et al*. temperature suitability model [18] is detailed.

### Satellite imagery and pre-processing

The primary temperature datasets used in this analysis were daytime and night-time Land Surface Temperature (LST), measured at an approximate spatial resolution of 1 × 1 km by the MODerate Resolution Imaging Spectroradiometer (MODIS) thermal sensor on board the NASA-Terra satellite system [28,29]. The daytime and nighttime measurements are associated with approximately 10:30 AM and 10:30 PM local time, respectively, governed by the overhead passing of the satellite. The raw data (MOD11A2) were acquired in tiles, 48 of which were used to create mosaics encompassing mainland Africa, Madagascar, and the smaller islands nations of São Tomé and Príncipe, Equatorial Guinea, Comoros, Mayotte, and some of the islands comprising the Seychelles. The temporal resolution of the selected MODIS LST products is 8 days, derived for each pixel as an average of between two and eight measurements [30] after omitting any with poor data quality (e.g., those capturing clouds). The resulting data archive consisted of 1,180 8-day composite mosaics (i.e. 590 each for daytime and nighttime LST), and spanned from day 65 of the year 2000 (i.e., March 5^th^, 2000) through to the end of 2012. 2From this archive, three composite dates (day 225 of year 2000, day 177 of 2001, and day 81 of 2002) were removed, as they contained no data or excessive error.

An important challenge for multi-temporal satellite imagery from tropical regions is the robust handling of errors caused by persistent cloud cover. Cloud obscures the sensor’s view of the Earth surface and leads to biased or missing temperature readings. To overcome the problem of missing data, the eight-day LST composites were first gap-filled using the method explained in Weiss *et al.* (personal communication with Weiss DJ, Atkinson PM, Bhatt SJ, Mappin BJ, Hay SI, Gething PW). Briefly, this method fills missing pixel values based on the values of usable pixels in close proximity and an adjustment that incorporates spatial patterns in LST present on anniversary dates (i.e., composite images from the same day, but different year) or from summarized datasets. The gap filling approach was highly accurate (Table 1) for LST, as demonstrated by introducing artificial gaps, filling them using the newly developed method, and then testing the resulting values against the original measurements. For the validation procedure, gaps were introduced as horizontal and vertical stripes with widths of (a) 25 pixels (i.e., ~25 km at the equator) to approximate the mean gap sizes present in the least gap-filled datasets, and (b) 500 pixels (i.e., ~500 km at the equator) to approximate the maximum gap sizes found in typical 8-day MODIS composites for Africa. The Root Mean Squared Error (RMSE) provides a summary indication of how well the gap-filling model is likely to perform for a single pixel.

**Table 1:** Validation results for the gap-filling procedure applied to the LST mosaics

**Year**	**Day of year**	**Calendar date**	**Type**	**Introduced stripe width (km)**	**R**^**2**^	**RMSE (degrees C)**
2001	145	May 25^th^	Night	25	0.976	0.750
2001	145	May 25^th^	Night	500	0.896	1.335
2004	65	Mar 5^th^	Night	25	0.974	0.865
2004	65	Mar 5^th^	Night	500	0.919	1.376
2006	177	Jun 26^th^	Night	25	0.980	0.890
2006	177	Jun 26^th^	Night	500	0.910	1.647
2011	321	Nov 17^th^	Night	25	0.978	0.895
2011	321	Nov 17^th^	Night	500	0.916	1.511
2012	241	Aug 28^th^	Night	25	0.969	0.838
2012	241	Aug 28^th^	Night	500	0.877	1.516
2001	129	May 9^th^	Day	25	0.983	1.179
2001	129	May 9^th^	Day	500	0.944	1.942
2005	257	Sep 14^th^	Day	25	0.978	1.142
2005	257	Sep 14^th^	Day	500	0.900	2.167
2005	9	Jan 9^th^	Day	25	0.973	1.392
2005	9	Jan 9^th^	Day	500	0.895	2.429
2006	73	Apr 14^th^	Day	25	0.967	1.273
2006	73	Apr 14^th^	Day	500	0.890	2.003
2007	145	May 25^th^	Day	25	0.983	1.204
2007	145	May 25^th^	Day	500	0.942	1.976
Mean			Night	25	0.975	0.848
Mean			Night	500	0.904	1.477
Mean			Day	25	0.977	1.238
Mean			Day	500	0.914	2.103

Validation datasets were selected at random, but stratified to capture a wide range of seasonal conditions.

### Association of LST and air temperature

The temperature constraint equations used within the Gething *et al*. temperature suitability model [18] were calibrated in experimental settings based on air temperature as opposed to LST. While LST is readily available in high resolution spatiotemporal datasets, air temperature is more closely aligned with adult vector survivorship and the duration of sporogony [19]. Although closely related physically, the relationship between the two metrics is complex and geographically heterogeneous. As such, a means of converting LST to air temperature was required to use the established temperature suitability modeling framework.

Numerous studies have explored the relationship between air temperature and LST measured from thermal sensors on satellites, including those onboard the MODIS platforms. A common finding is a strong linear correlation (i.e., *R*^2^ > 0.8) between night-time LST and minimum air temperatures, but a less predictable relationship between daytime LST and maximum air temperatures e.g., [31]. This discrepancy has been the subject of modeling efforts that utilized the relationship established by Nemani and Running [32] between LST and vegetation indices derived from satellite imagery as a correction factor [33,34]. The basic vegetation index correction concept has since been extended using factors such as soil moisture [35], elevation [36] and, most recently, a wide suite of land-cover variables in a multivariate mixed-effects statistical model [19]. Alternative methods have also been devised such as a correction based on solar zenith angle [37]. However, despite these efforts, no consensus exists on the optimum approach for converting LST to air temperature, particularly for continental-to-global scale analyses in which land cover variability and seasonality greatly affect the relationship between daytime LST and maximum air temperature [38].

For the present study, a parsimonious land-to-air temperature conversion model was sought that could provide accurate conversions without requiring complex handling of ancillary land cover data. This was driven by the very large computational demands associated with processing the high-resolution spatiotemporal data cube, and the difficulties associated with obtaining reliable temporally-dynamic data on putative land cover covariates. To devise an alternative approach, a dataset of georeferenced air temperature observations was required that could be combined with MODIS LST values to investigate the resulting bivariate relationships. Daily data was obtained for all meteorological stations available within Africa from the NOAA National Climatic Data Center consisting of daily minimum and maximum temperatures for 407,857 daily observations from 154 meteorological stations. These data were summarized to create 8-day estimates for minimum and maximum temperature that matched the averaging periods associated with the LST dataset. After removing any with fewer than three daily observations for minimum or maximum temperature, the final number of 8-day temperature observations was 12,531.

**Figure 1:**
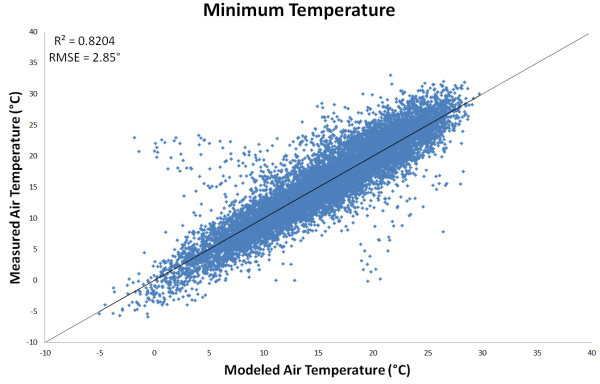
**Comparison of observed versus modelled minimum air temperature (*T***_**max**_**).** The model was based on nighttime land-surface temperature (LST_night_) from the MODIS sensor.

As reported by other authors [31], a strong linear relationship (*R*^2^ > 0.82) between minimum air temperatures (*T*_min_) and night-time LST (*LST*_night_) was found, which negated the need to include additional variables when converting *LST*_night_ to *T*_min_. Instead a modest correction (i.e., a linear model with intercept close to zero and gradient close to one, Equation 1) was applied to marginally improve the RMSE. The result of the *T*_min_ model is shown in Figure 1.

(1) $$T_{\text{min}}\;=\;0.209\;+\;0.971\;˙\;\mathrm{LS}T_{\mathrm{night}}$$

**Figure 2:**
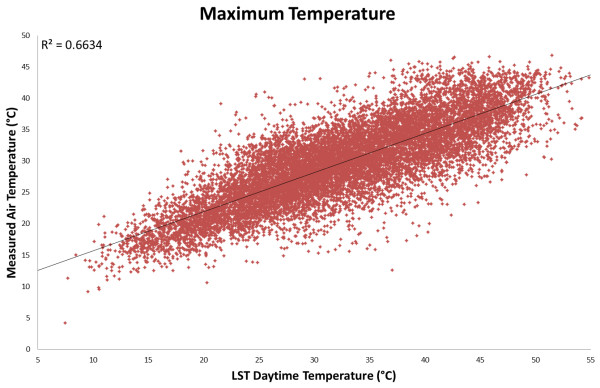
**Comparison of maximum air temperature (*T***_**max**_**) observed at ground stations and daytime land-surface temperature (*LST***_**day**_**) measured by the MODIS sensor.**

**Figure 3:**
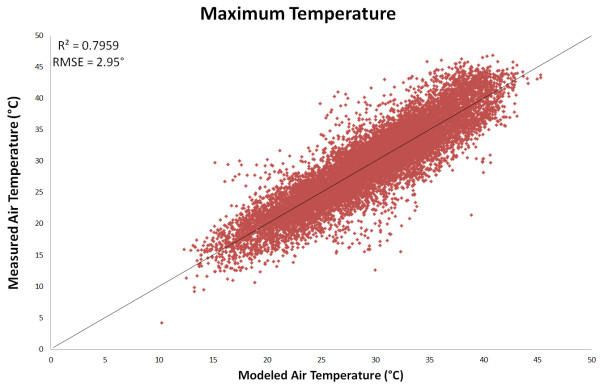
**Comparison of observed versus modelled maximum air temperature (*T***_**max**_**).** The model was based on daytime land-surface temperature (LST_day_) and daily temperature range (LST_Δ_) from the MODIS sensor, along with the number of daylight hours DAY_length_ at each location.

The linear relationship between daytime LST (*LST*_day_) and maximum air temperature (*T*_max_) was weaker (R^2^ < 0.67, Figure 2) than that between *LST*_night_ and *T*_min_. To augment this model, the inclusion of the diurnal temperature range, *LST*_Δ_ = *LST*_day_ − *LST*_night_, was investigated. This follows from studies [39,40] that found the difference between satellite-derived maximum and minimum LST is inversely related to both moisture availability and thermal inertia properties of the land surface. Further work [32] has demonstrated how those same properties mediate the relationship between land surface and air temperature by comparing LST measurements for a single forest patch, on days with very similar air temperature and relative humidity, but with markedly different moisture availability. In this study [32], when the forest patch was moist, LST was lower and more similar to the measured air temperature than during the dry period, which was likely the product of latent heat flux (i.e., thermal energy lost from the surface due to evapotranspiration). Together these findings suggest that cells with high *LST*_Δ_ will be associated with lower moisture availability and therefore a greater disparity between daytime LST and *T*_max_. Including *LST*_Δ_ in the *T*_max_ model resulted in an improvement in *R*^2^ from 0.66 (AIC 68952) to 0.78 (AIC 63389). Additional variables tested for their utility in modeling *T*_max_ were latitude, longitude, Enhanced Vegetation Index (EVI), elevation, and daylight hours as calculated using the approach defined by Forsythe *et al*. [41]. No variables other than *LST*_Δ_ increased *R*^2^ to above 0.70. However, when daylight hours (*DAY*_length_) was then included as a third covariate the overall model performance improved significantly (*R*^2^ of 0.80, AIC 62688), albeit more modestly than through the inclusion of *LST*_Δ_ alone. It is plausible that daylight length improves the model as it captures the time that elapses between sunset and the night-time passing of the MODIS sensor (i.e., at 10:30 PM local time), which is relevant for *LST*_Δ_ since the land surface will have less time to cool when the sun sets later. None of the other variables tested warranted inclusion in the final model as they were either non-significant or had very little effect on model performance. Therefore, the final *T*_max_ model used *LST*_day_, *LST*_Δ_, and *DAY*_length_ as predictor variables (Equation 2) and produced a final *R*^2^ for *T*_max_ similar to that for *T*_min_ (Figure 3).

(2) $$T_{\text{max}}\;=\;8.149\;+\;\mathrm{LS}T_{\mathrm{day}}\;\;˙\;0.949\;\mathrm{LS}T_{\Delta }\;\;˙\;-\;0.541\;+\;\mathrm{DA}Y_{\mathrm{length}}\;\;˙\;-\;0.866$$

### Temperature interpolation

As in the original Gething *et al*. [18] model, it was necessary to interpolate the 8-day *T*_min_ and *T*_max_ data time-series at each pixel to a much finer temporal resolution to incorporate diurnal variation and allow a 2-hour discrete time-step in the temperature suitability model, representing an acceptable approximation of a continuous process. A two-step interpolation process was implemented and applied to each pixel for each monthly processing step by first creating daily *T*_min_ and *T*_max_ values from the 8-day composites and then converting the daily temperature values into 2-hour slices by adjusting for diurnal temperature variability. The first step in this process was to identify all 8-day LST periods that intersected the temporal window of the month in question plus the 31 preceding days, and then fitting a spline through the 8-day *T*_min_ and *T*_max_ values to produce daily estimates. With daily *T*_min_ and *T*_max_ values, the day of the year of each day, and the latitude and longitude of the pixel, the diurnal temperature curve fitting approach used by Garske *et al*. [19] was applied, which modified daytime temperature using a sine wave model (Equation 3) and nighttime temperatures using an exponential decay function (Equation 4). For these equations the times of sunrise and sunset were calculated using the Office of the United States Naval Observatory algorithm [42].

(3) $$\begin{array}{l} T\;\left(t\right)\;=\;T_{\text{min}}\;+\;\left(T_{\text{max}}\;-\;T_{\text{min}}\right)\;˙\;\sin\;\left(\pi \frac{t\;-\;t_{\mathrm{sunrise}}}{\left(t_{\mathrm{sunrise}}\;-\;t_{\mathrm{sunrise}}\right)\;+3.72}\right)\\ \;\text{for}\;\text{day}\;\text{time}\;\text{temperatures} \end{array}$$

(4) $$\begin{array}{l} T\;\left(t\right)\;=\;T_{\text{min}}\;+\;\left(T_{\mathrm{sunset}}\;-\;T_{\text{min}}\right)\;˙\;\exp\;\left(‒2.2\frac{t\;-\;t_{\mathrm{sunset}}}{24\;-\;\left(t_{\mathrm{sunset}}\;-\;t_{\mathrm{sunrise}}\right)\;}\right)\\ \;\mathrm{for}\;\mathrm{nigthttime}\;\mathrm{temperatures} \end{array}$$

### Temperature suitability model for ***Plasmodium falciparum***

The temperature suitability model defined in Gething *et al*. [18] was implemented using the derived 13-year, two-hour resolution air temperature time-series for each 1 × 1 km pixel. That model is described at length in the earlier study [18] and, therefore, the full description is not repeated here. In brief, the model captured two key mechanisms by which temperature mediates the transmission cycle of *P. falciparum*: survival of adult *Anopheles* and the extrinsic incubation period of the parasite within the vector. Note that the selected temperature suitability model does not currently incorporate the effect of temperature on larval development, which impacts the density of adult mosquitoes and may affect epidemiologically important aspects of mosquito biology. The model simulated for every 1 × 1 km pixel a new cohort of vectors (of arbitrary size) emerging every two hours throughout the modeled period. It then tracked for each cohort their proportional survival [43] and capacity to support a complete extrinsic incubation period [44] as functions of the continuously changing temperature regime at each location. Practically speaking, each two-hour time step was intersected by 372 cohorts (i.e., all the cohorts that emerged from the preceding 31 days), and if the cohort was infectious at the intersecting time step, the proportion of surviving mosquitoes was added to the cumulative temperature suitability total for that time step. Intuitively, a newly emerged cohort immediately begins to decline in size as individual mosquitoes die (according to a temperature dependent daily survival probability). Simultaneously, that cohort can take blood meals, become infected, and the ingested gametocytes can begin their development into mature sporozoites, again according to a temperature dependent process. By capturing this process numerically for each cohort, and integrating across all cohorts present in each pixel at each time step, the model evaluated the relative abundance of vectors, and the proportion that are potentially infectious, at all space-time locations. The resulting pixel-level time-series of the proportion of infectious vectors were then aggregated temporally and standardized by the maximum possible predicted value, leading to a comparable Temperature Suitability Index (TSI) value between zero and one for every pixel in each month.

## Results

**Figure 4:**
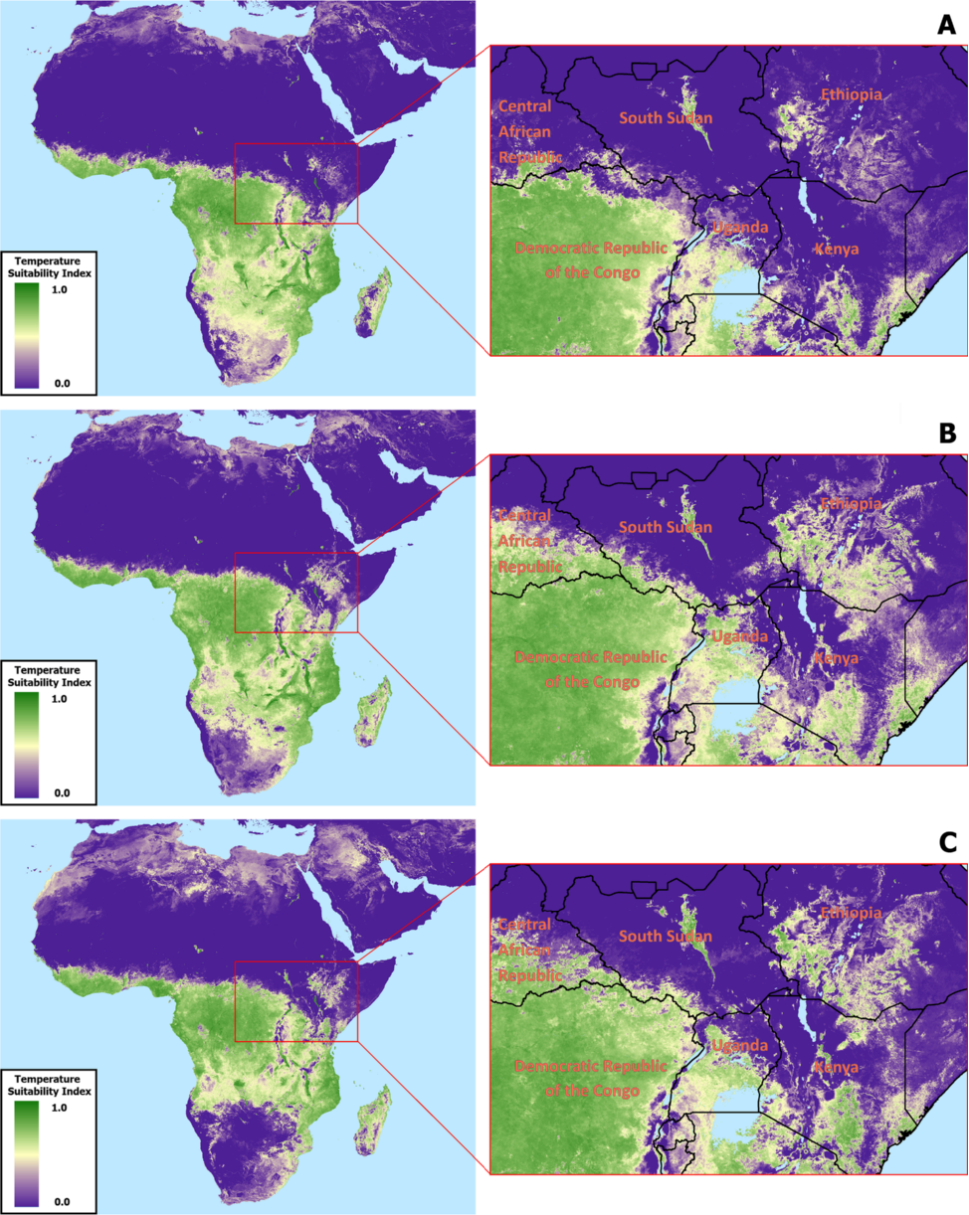
**Example temperature suitability index predictions for three consecutive Aprils.** Shown are **(A)** April 2000, **(B)** April 2001 and **(C)** April 2002. Note the obvious differences in southern Africa and Madagascar, as well more subtle differences visible in the zoomed-in view of East Africa (e.g., southern Central African Republic as well as the Ethiopian highlands).

A total of 154 monthly grids of TSI were produced, spanning the period of April 2000 through December 2012. An animation showing the full set of monthly TSI rasters is available for download as Additional file 1. The results show spatial patterns similar to average monthly conditions derived in previous analyses [18,19], but the utility of the new dataset is most apparent in its ability to characterize inter-annual variability. For example, Figure 4A – 4C illustrate noticeable differences in modeled TSI for the month of April in southern Africa, Madagascar, and to a lesser extent in east Africa (highlighted in the zoomed-in portion of the figures). All other months in the dataset show similar spatiotemporal variability when compared across different years in areas experiencing transitional seasons (e.g., in months during which changes from warm to cool seasons occur).

**Figure 5:**
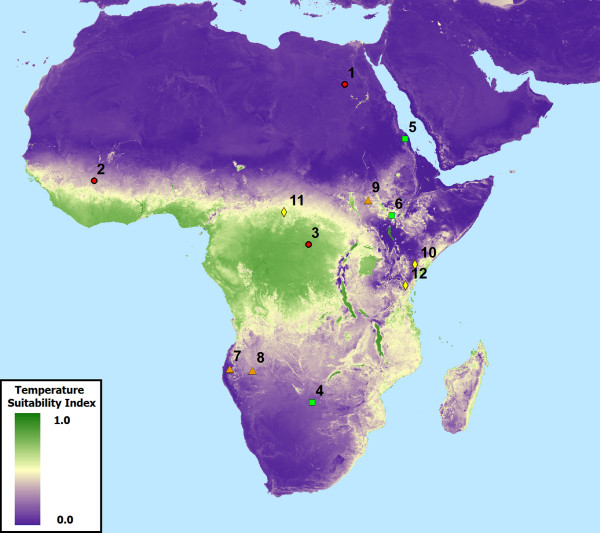
**Comparison points for demonstrating the utility of dynamic TSI over synoptic products.** Mean annual TSI is used here as the background dataset.

**Figure 6:**
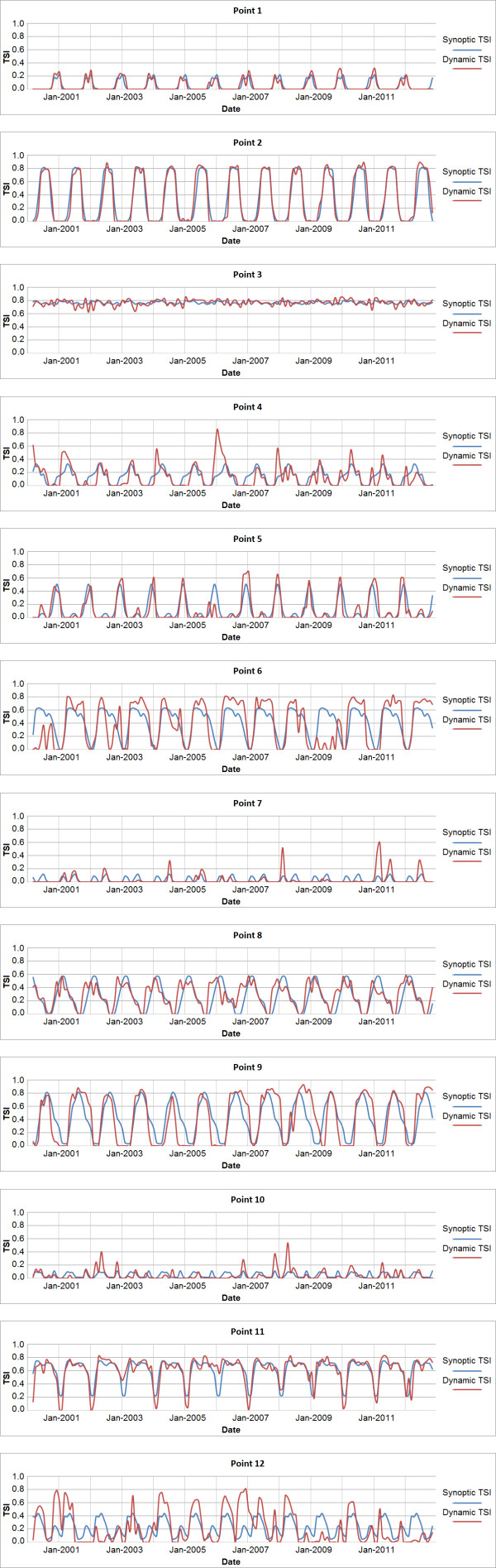
**Longitudinal comparison of dynamic vs. synoptic TSI.** Points 1 - 3 are cases where the dynamic TSI data provide limited improvement over synoptic averages. Points 4 - 12 are examples of areas where the magnitude and/or timing of TSI are poorly represented by synoptic data in one or more years.

**Figure 7:**
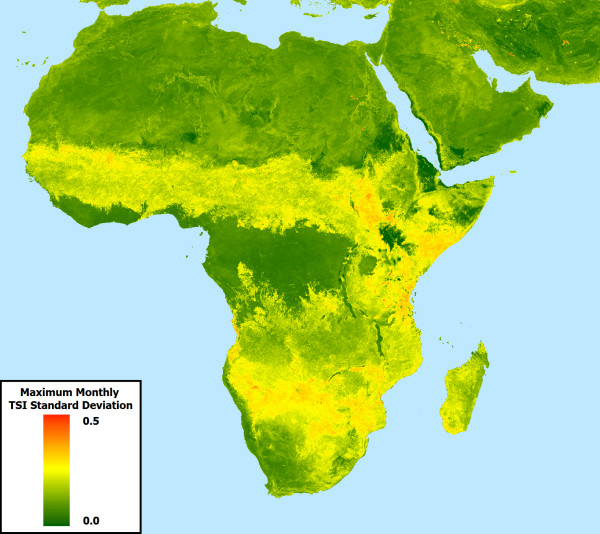
**Maximum monthly TSI standard deviation.** The value represented by each cell is the maximum per-month standard deviation, as determined from the value calculated for each month (Jan. – Dec.) from the 13-year longitudinal dataset (2000-2012). The month of greatest standard deviation is typically associated with seasonal transitions such as spring, fall, or the onset or conclusion of the wet season.

To further illustrate the utility of the dynamic temperature suitability dataset, longitudinal graphs were created for randomly distributed points (n = 50), twelve of which are shown in Figure 5 to highlight how synoptic data may fail to adequately capture variability within malaria temperature suitability (Figure 6). Generally speaking, areas that experience seasonal variability in TSI can be characterized much more thoroughly through the use of a dynamic product than with a synoptic product, as the timing, magnitude, and duration of TSI peaks and troughs vary from year to year. In cases where TSI is always low, such as in the Sahara, or always high, such as in west African equatorial rain forests, synoptic TSI is functionally equivalent to the new dynamic TSI. This is also true in rare cases where intra-annual TSI patterns are highly consistent from year to year. However, the vast majority of areas with endemic malaria that experience seasonal variability in TSI have at least one month during year in which TSI (which ranges between 0.0 and 1.0) has a standard deviation between approximately 0.1 and 0.25, with some areas exceeding 0.4 (Figure 7).

## Discussion

As malaria mapping and research projects are adapted and expanded to include both space and time, dynamic spatiotemporal data products will be essential for modeling changing malaria infection prevalence and disease burden. This work creates one such dynamic covariate, building on the earlier, static, product established by Gething *et al*. [18] which subsequently proved the most informative covariate in the contemporary modeling of global *P. falciparum* malaria [15]. Ultimately, only downstream modeling efforts will fully demonstrate the benefit of dynamic TSI over synoptic products, but given the inter-annual variability evident within the dynamic TSI product, this dataset is expected to significantly improve the predictive capacity of malaria risk models.

The dynamic TSI dataset is particularly useful for capturing infrequent events that interrupt typical seasonal patterns of malaria transmission. Noteworthy examples of such events are (1) peaks in TSI that are higher or last longer than TSI peaks in a typical year, and (2) troughs in TSI that are smaller (or non-existent) or shorter than usual. Furthermore, in areas where the timing of seasonal transitions is irregular, synoptic data created from simple averaging will have an unrealistically low amplitude in cases where peaks or troughs can occur in different months (e.g., if peaks occur in either January or February the resulting synoptic average will have a peak spanning both month, but not as high as an actual peak due to the muting effect of averaging). From a malaria control perspective, areas that experience unusually high or long TSI peaks may experience outbreaks within human populations that have low acquired functional immunity or occur in areas poorly equipped to deal with an outbreak (e.g., without adequate stores of anti-malarial drugs) due to the ephemeral nature of local malaria infection periods [45]. Even in areas accustomed to seasonal malaria [46], unusual timing or durations of periods of transmission suitability have implications for malaria commodity procurement, control planning, and implementation.

## Conclusions

Creation of dynamic temperature suitability products is the logical progression from previous research endeavors that established a methodology for creating synoptic products, as well as a necessary step for developing spatiotemporal malaria prevalence and burden models that utilize temperature suitability as a predictor variable. The datasets underlying the TSI data product resulting from this research are daily minimum and maximum temperatures, which were modeled from MODIS LST datasets using an approach described in this paper. The use of temperature data from MODIS facilitated the creation of output with a 1 × 1 km spatial resolution, which represents the highest spatial resolution malaria temperature suitability product currently available for Africa. The temporal resolution and extent of the MODIS data archive, in combination with substantial advances in algorithmic efficiency, allowed TSI to be modeled monthly from April 2000 through December 2012. With the operational TSI production methodology established by this research, data continuity can be maintained for the Africa TSI product for the life of MODIS sensor, as well as adapted for use with LST data produced from alternate sensors. Furthermore, the spatial scope of the TSI product can be expanded to include other areas of the tropics with malaria transmission, including those with endemic *Plasmodium vivax*, and potentially be adapted for use with other vector-borne diseases affected by temperature.

## Competing interests

The authors declare that they have no competing interests.

## Authors’ contributions

DJW improved upon the previous temperature suitability algorithm, adapted the new algorithm for use with dynamic data, acquired and gap-filled all LST data using an approach described in another publication, developed the approach for modeling minimum and maximum air temperature from LST data, and drafted and revised the manuscript. SB revised the manuscript. BM revised the manuscript. TVPB is a co-developer of the biological model for synoptic malaria temperature suitability, and revised the manuscript. DLS is a co-developer of the biological model for synoptic malaria temperature suitability and revised the manuscript. SIH revised the manuscript. PWG is a co-developer of the biological model for synoptic malaria temperature suitability, contributed to the theoretical development of the dynamic TSI algorithm, and acted as primary reviser of the manuscript. All authors read and approved the final manuscript.

## Supplementary Material

Additional file 1 This animation iterates through the full set of 154 monthly TSI rasters resulting from this research. Click here for file
